# Genome-Wide Identification and Expression Analysis of the *14-3-3* Gene Family in Mango (*Mangifera indica* L.)

**DOI:** 10.3390/ijms23031593

**Published:** 2022-01-29

**Authors:** Liming Xia, Xinhua He, Xing Huang, Haixia Yu, Tingting Lu, Xiaojie Xie, Xuemei Zeng, Jiawei Zhu, Cong Luo

**Affiliations:** State Key Laboratory for Conservation and Utilization of Subtropical Agro-Bioresources, College of Agriculture, Guangxi University, 100 East Daxue Road, Nanning 530004, China; xialimingguangxi@163.com (L.X.); honest66222@163.com (X.H.); xinghuangkeys@163.com (X.H.); yuhaixia0201@163.com (H.Y.); lutting4040@163.com (T.L.); xiexiaojie0827@126.com (X.X.); zengxuemei1228@163.com (X.Z.); zhujiaweiii1206@163.com (J.Z.)

**Keywords:** mango, *14-3-3* gene family, genome-wide identification, expression characteristics

## Abstract

Members of the *Mi14-3-3* gene family interact with target proteins that are widely involved in plant hormone signal transduction and physiology-related metabolism and play important roles in plant growth, development and stress responses. In this study, *14-3-3s* family members are identified by the bioinformatic analysis of the mango (*Mangifera indica* L.) genome. The gene structures, chromosomal distributions, genetic evolution, and expression patterns of these genes and the physical and chemical properties and conserved motifs of their proteins are analysed systematically. The results identified 16 members of the *14-3-3* genes family in the mango genome. The members were not evenly distributed across the chromosomes, and the gene structure analysis showed that the gene sequence length and intron number varied greatly among the different members. Protein sequence analysis showed that the Mi14-3-3 proteins had similar physical and chemical properties and secondary and tertiary structures, and protein subcellular localization showed that the Mi14-3-3 family proteins were localized to the nucleus. The sequence analysis of the *Mi14-3-3s* showed that all Mi14-3-3 proteins contain a typical conserved PFAM00244 domain, and promoter sequence analysis showed that the *Mi14-3-3* promoters contain multiple hormone-, stress-, and light-responsive cis-regulatory elements. Expression analysis showed that the *14-3-3* genes were expressed in all tissues of mango, but that their expression patterns were different. Drought, salt and low temperature stresses affected the expression levels of *14-3-3* genes, and different *14-3-3* genes had different responses to these stresses. This study provides a reference for further studies on the function and regulation of *Mi14-3-3* family members.

## 1. Introduction

The *14-3-3s* family is composed of proteins encoded by multiple genes (including multiple isomers encoded by homologous genes) that are found in a wide variety of eukaryotes. 14-3-3 proteins promote or inhibit the activities of various enzymes, serve as bridges for protein–protein interactions, and regulate cell protein levels and locations. 14-3-3 proteins, which are important regulatory proteins, also play crucial roles in the signalling of several hormones involved in normal plant-development-related metabolism and stress responses [1]. In recent years, researchers have made breakthroughs concerning the involvement of 14-3-3 proteins in the regulation of flower formation, abiotic stress responses, energy metabolism, and signal transduction in plants, and 14-3-3 proteins constitute a popular research topic in the field of plant signalling.

*14-3-3s* are involved in both the photoperiod and the gibberellin (GA) pathways in plants. The florigen homologue *SFT* in tomato has been found to interact with 14-3-3 proteins [2]. The overexpression of *PvGF14b*, *PvGF14c* and *PvGF14e* from *Phyllostachys violascens* significantly delays the flowering time of transgenic *Arabidopsis thaliana*. These results show that at least three *PvGF14* genes are involved in the flowering network of bamboo; in addition, through its interaction with *FLOWERING LOCUS T* (*PvFT*), *PvGF14c* has been found to act as a negative regulator of flowering in *Phyllostachys violascens* [3]. In the cytoplasm of rice, Hd3a interacts with *14-3-3s*, and the *Hd3a-14-3-3* complex interacts with a rice *FLOWERING LOCUS D* (*FD*) homologue, *OsFD1*, to form a heterohexameric protein complex called the florigen-activating complex (*FAC*). The interaction of *TERMINAL FLOWER 1-like* (*TFL1*) with *FD* and *14-3-3* binding sites suggests that *TFL1* creates a similar compound (*FRC*) to inhibit *FT*. In the meristem, *RCN* can interact with *14-3-3s* and *OsFD1*, and four *RICE CENTRORADIALIS* genes (*RCNs*) interact with *GF14b*, *GF14c*, *GF14e* and *GF14f*. However, *Hd3a* does not interact directly with *OsFD1* in vitro, so 14-3-3 proteins mediate the interaction between *Hd3a* and *OsFD1* [4,5,6].

Increasingly, in-depth studies on the functions of 14-3-3 proteins have revealed that these proteins play roles in plant responses to drought, salt and cold stresses by regulating stomatal opening, root movement, signalling hormones, physiology-related metabolism and stress responses [7,8,9,10,11]. Although 14-3-3 proteins in plants have a highly conserved target-binding domain, several studies have shown that different *14-3-3* subtypes can modulate different targets at different locations under different abiotic stresses [12]. In rice, 14-3-3 proteins regulate the rice glycometabolism pathway by interacting with *MYBS2* to regulate α-amylase (α-amy) in the nuclear cytoplasmic transport pathway [13]. *OsGF14c*, a *14-3-3* subtype, positively regulates the drought resistance of rice seedlings. *OsGF14b* negatively regulates drought resistance by altering drought-stress-related parameters (peroxidase activity and malondialdehyde (MDA), proline and soluble sugar levels) [14,15]. The overexpression of *GF14c* in rice results in delayed flowering and early flowering in a functional deletion (T-DNA insertion) mutant, suggesting that *GF14c* is a flowering suppressor [16]. The downstream MADS-box transcription factor 15-like (*OsMADS15*) gene is then activated to induce flowering in rice [17]. Under salt stress, the *GhGRF3*, *GhGRF4*, *GhGRF5*, *GhGRF7* and *GhGRF16* genes have been found to be downregulated in the leaves of cotton [18].

To date, different numbers of *14-3-3* genes have been identified in a variety of plant species, including 13 in Arabidopsis [19], 8 in rice [20], 16 in soybean [21], 17 in tobacco [22], 9 in poplar [23], 18 in apple [24] and 8 in sweet orange [25]. Until now, however, little information has been available about *14-3-3* family members in mango. Mango is an important tropical and subtropical fruit tree species that is affected by various environmental factors during its growth and development. Plant 14-3-3 proteins are involved in protein–protein interactions and play important roles in diverse biological processes. In this study, we identify sixteen *Mi14-3-3* gene family members from a mango genome database and analysed their physical and chemical properties, chromosomal locations, gene structures, evolutionary relationships, promoter cis-regulatory elements and expression profiles in different tissues and in response to low temperature, drought and salt stress treatments. Our aim is to lay a foundation for future studies of the functions of the *Mi14-3-3* genes in mango-flowering-regulation and stress responses.

## 2. Results

### 2.1. Genome-Wide Identification of Mi14-3-3 Gene Family Members and Characterization of Their Proteins

Using the protein sequence of an Arabidopsis 14-3-3 protein family member as bait, we evaluated the conserved domain of the target sequences by bioinformatic analysis and removed the redundant protein sequences with HMMER and SMART online software. Sixteen members of the *Mi14-3-3* family were identified from the ‘Sijimi’ mango genome (unpublished) (Table 1), which is consistent with the number of 14-3-3 proteins found in the published genome of mango (Alfonso) [26]. The lengths of the *14-3-3* genes in mango ranged from 747 to 840 bp, the numbers of encoded amino acids ranged from 247 (*Mi14-3-3-C2*) to 278 (*Mi14-3-3-I1*) amino acids, and the molecular weights (MWs) ranged from 28.08 kD (*Mi14-3-3-C2*) to 30.97 kD (*Mi14-3-3-I1*). The results showed that the isoelectric points (pIs) of the Mi14-3-3 proteins were less than 7.0, which indicated that the proteins were acidic, and the grand average of hydropathy (GRAVY) of the Mi14-3-3 proteins was negative, which indicated that the proteins were hydrophilic. The instability indices of all Mi14-3-3 proteins were higher than 40.

**Figure 1:**
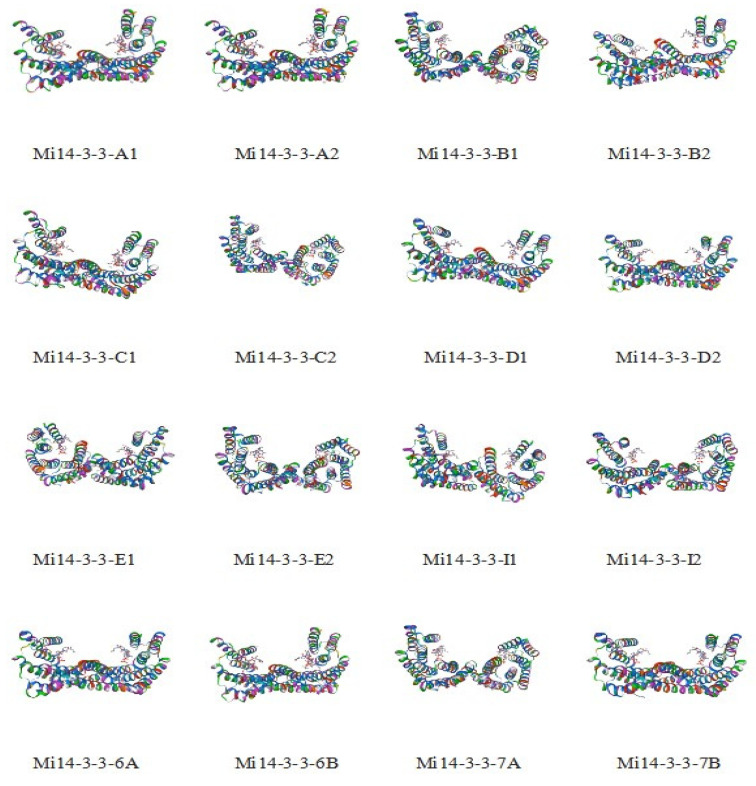
Tertiary structures of Mi14-3-3 proteins.

The secondary structures of Mi14-3-3 family proteins were analysed. The secondary structure of a protein is the specific conformation achieved when the backbone atoms of a polypeptide chain are coiled or folded along a certain axis—in other words, the spatial arrangement of atoms in the backbone of a peptide chain (the side chains of the amino acid residues are not involved). These structures generally include α-helices, β-folds, β-turns, and random crimps. The secondary structures of the 16 Mi14-3-3 proteins were analysed with SOPMA software (Table S1). The results showed that the Mi14-3-3 proteins had similar secondary structures, indicating that these proteins may form similar higher-order structures to perform similar functions. The proportion of α-helices was the highest (65.28%~3.81%), followed by the proportion of random coils (20%), while the proportions of extended strands and β-turns were the lowest. β-Turns are common stable secondary structures in polypeptide chains that mainly connect α-helices and β-folds in proteins. Among the 16 14-3-3 proteins of mango, *Mi14-3-3-6A* was found to comprise 3.07% β-turns, and *Mi14-3-3-I2* was found to comprise 0.75% β-turns. It is speculated that *Mi14-3-3-6A* may have more changes in the direction of polypeptide chains and a more complex structure than *Mi14-3-3-I2*. The tertiary structure of a protein is formed by further winding and folding on the basis of the secondary structure and is mainly maintained by hydrophobic interactions between amino acid side chains, hydrogen bonds and electrostatic interactions. To elucidate the mechanism of action of a protein, it is necessary to predict the three-dimensional structure of the protein. Therefore, we used an online software to predict the tertiary structures of the 16 14-3-3 protein family members and found that the proteins that have similar three-dimensional conformations are composed mostly of α-helices and have symmetrical spatial structures (Figure 1).

### 2.2. Multiple Sequence Alignment of Mi14-3-3 Proteins

**Figure 2:**
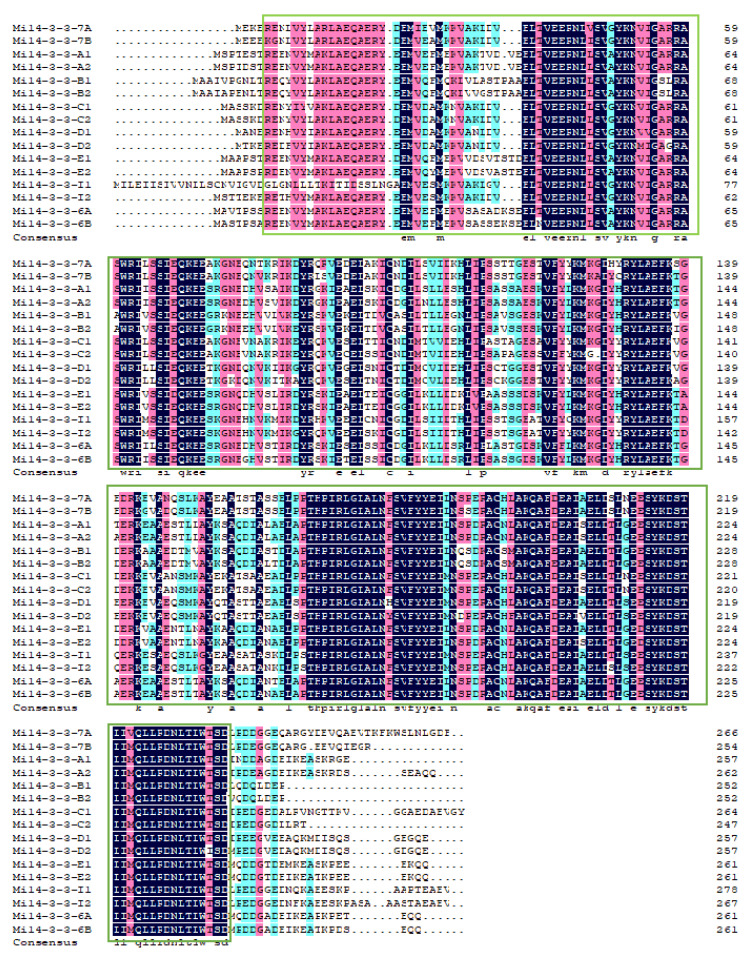
Multiple sequence alignment of all 14-3-3 proteins in mango. The typical conserved PFAM00244 domain is marked by a green box.

The multiple sequence alignment of the Mi14-3-3 protein family members via DNAMAN software (Figure 2) showed a high degree of similarity among them, and all of the proteins were found to have a PFAM00244 *14-3-3* superfamily domain. These results show that the structures of the proteins in this family are highly conserved.

### 2.3. Gene structure and Motif Analysis

**Figure 3:**
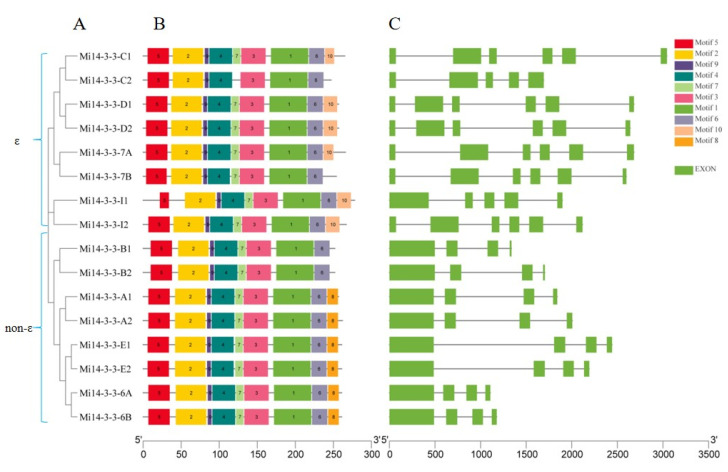
Phylogenetic relationships, gene structure and conserved motifs of the *Mi14-3-3* genes. (**A**) Construction of a rootless neighbour-joining phylogenetic tree comprising 16 *Mi14-3-3* gene sequences. (**B**) Distribution of conserved motifs within the *14-3-3* gene sequences. The differently coloured boxes represent different bases, and the motif numbers of the genes are shown in the coloured boxes. (**C**) Exon/intron structures of *Mi14-3-3* genes. The green boxes represent exons, and the same-length black lines represent introns. The lengths of the exons can be inferred from the scale at the bottom.

To systematically analyse the gene structures of *Mi14-3-3* genes, a *14-3-3* gene structure map (Figure 3A,B) was constructed with TBtools. Exon/intron pattern differences play crucial roles in evolution. We analysed the exon/intron maps of the *Mi14-3-3* genes and found that they contained 3–5 introns. The non-ε class *Mi14-3-3* gene contained 3 introns, while the ε class genes contained 4–5 introns. The exon/intron patterns of the *Mi14-3-3* genes were significantly different between the two groups, reflecting the diversity of *Mi14-3-3* genes that has arisen during evolution (Figure 3A, Table 1). A total of 10 conserved motifs of Mi14-3-3 gene family proteins were predicted by MEME, among which motif 5, motif 2, motif 9, motif 4, motif 3, motif 1, and motif 6 were conserved in ε class and non-ε class *Mi14-3-3* genes and were the characteristic motifs of *Mi14-3-3s*. The C-terminal motif, which may be responsible for the different target proteins, had high variability among the members of the two subfamilies; ε class *14-3-3* genes mainly contained a specific motif 10 domain, while the non-ε class genes contained specific motif 8 and motif 7 domains (Figure 3C).

### 2.4. Chromosome Distribution of 14-3-3 Gene Family Members in Mango

**Figure 4:**
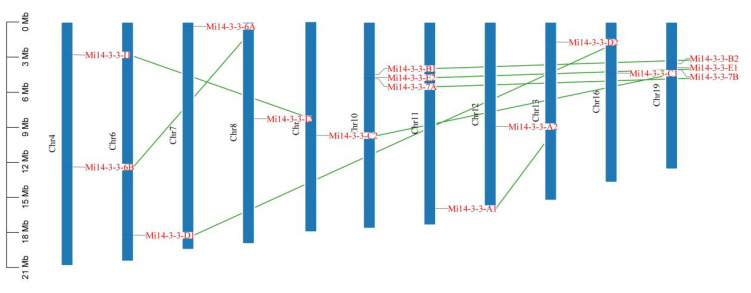
Genomic distribution of *14-3-3* (*Mi14-3-3*) genes across the eleven mango chromosomes. The list of organisms by chromosome count is shown on the upper-left side of each chromosome. The scale is in megabases (Mb). The chromosomal locations of the *Mi14-3-3s* were determined according to the physical location of each gene.

The positions of the *14-3-3* gene family members in the mango genome were analysed. The results showed that the 16 *Mi14-3-3* genes were heterogeneously distributed on 11 of the 20 chromosomes (Figure 4). They were most densely distributed on chromosomes 10 and 18, which had three genes each, while there were two genes on the second chromosome, and there was one on each of the other eight chromosomes. To better understand the evolution of the M*i14-3-3* genes, we examined the genome duplication events of this gene family. The *Mi14-3-3* gene pairs presented eight sets of repeat events, but no tandem repeats. Chromosomes 10 and 18 had the most repeat events.

### 2.5. Phylogenetic Analysis of the Mi14-3-3 Genes

**Figure 5:**
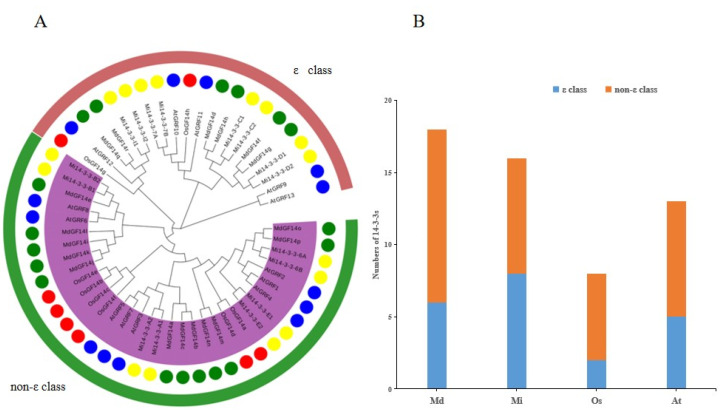
Phylogenetic analysis of *14-3-3* genes in *Mangifera indica* L., *Arabidopsis thaliana*, *Oryza sativa*, and *Malus domestica*. (**A**) The phylogenetic tree depicts the relationships among 16 *Mi14-3-3* (yellow circles), 8 *OsGF14* (red circles), 18 *MdGF14* (green circles) and 13 *AtGRF* (blue circles) genes. The rootless tree was constructed using the LG model of MEGA 11.0 and divided into two subfamilies. The different groups are marked with differently coloured branches, and the different background colours indicate the various *14-3-3* gene types. (**B**) Statistical analysis results for *14-3-3* members from apple (Md), mango (Mi), rice (Os) and Arabidopsis (At).

To explore the evolutionary relationships of the Mi14-3-3 proteins, *Arabidopsis thaliana 14-3-3* (*AtGRF*), *Oryza sativa 14-3-3* (*OsGF*) and *Malus domestica 14-3-3* (*MdGF*) genes were selected, and phylogenetic trees of mango, apple, rice and Arabidopsis *14-3-3* genes were constructed. As shown in Figure 5, the Mi14-3-3 genes were found to be very weakly related to the Arabidopsis *14-3-3* genes and were located on different branches. However, the Mi*14-3-3* genes were more closely related to the apple *14-3-3* genes, suggesting that *14-3-3* genes have been more conserved during the evolution of woody plant species than in the evolution of herbaceous plant species. Similar to the Arabidopsis and rice *14-3-3* gene families, the Mi*14-3-3* gene family could be divided into ε class and non-ε class types.

### 2.6. Analysis of the Cis-Regulatory Elements of Mi14-3-3 Gene Family Member Promoters

**Figure 6:**
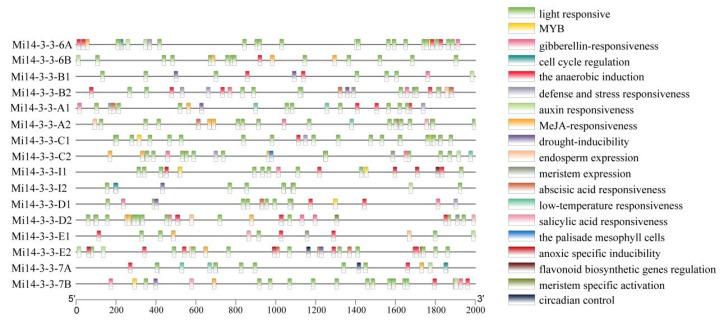
Visualization of the results of an analysis of cis-regulatory elements within the promoters of Mi14-3-3 gene family members.

To understand the expression and regulatory characteristics of the Mi*14-3-3* gene family members, we analysed the promoter sequence of each of these genes (the 2 kb region upstream of the coding region) via the PlantCARE database (Figure 6). The 16 *Mi14-3-3* promoter sequences contained 21 types of photoresponsive elements, suggesting that *Mi14-3-3* genes may be induced by many hormones and may participate in many plant physiological and metabolic processes. Most Mi*14-3-3* promoter regions contained a variety of stress-responsive elements, including LTR-, MYB-, ARE-, MBS- and TC-rich repeats. These results indicate that members of the Mi*14-3-3* family are regulated by multiple stress signals and may participate in specific growth/development, maturation/senescence, and stress-response pathways.

### 2.7. Synteny Analysis of Mi14-3-3 Genes

**Figure 7:**
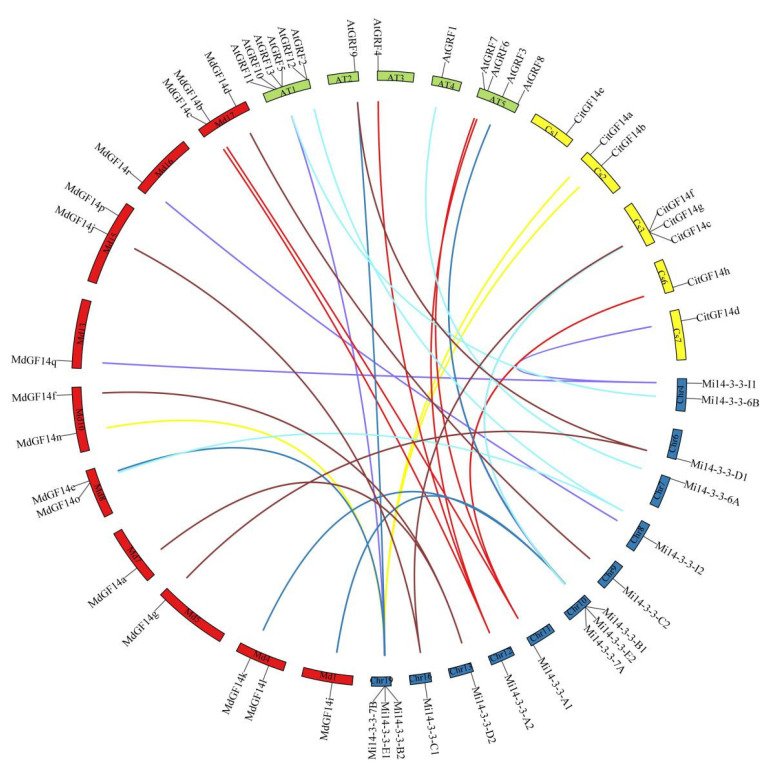
Syntenic relationships of mango, apple, *Citrus sinensis* and *Arabidopsis* 14-3-3 genes. The coloured curves represent mango (blue), apple (red), *Citrus sinensis* (yellow) and *Arabidopsis* (green) syntenic gene regions. The graph was generated via Circos.

The evolution and amplification of gene families are closely related to the occurrence of tandem repeats and segmental repeats. Tandem duplication usually refers to a gene cluster formed by multiple members of a family in the same intergenic region. The most common segmental duplication event in plants leads to additional family members on different chromosomes [27]. To understand the amplification pattern of the *Mi14-3-3* genes in the mango genome, we performed a collinearity analysis. The results showed that six *Mi14-3-3* genes (*Mi14-3-3-B1*, *Mi14-3-3-B2*, *Mi14-3-3-E1*, *Mi14-3-3-E2*, *Mi14-3-3-7A*, and *Mi14-3-3-7B*) were linked to form two tandem repeats on chromosomes 10 and 18. The *Mi14-3-3-6A/6B*, *Mi14-3-3-C1/C2*, *Mi14-3-3-D1/D2* and *Mi14-3-3-I1/I2* gene pairs may have been generated by fragment duplication because they were located on different homologous chromosomes (Figure 7), and the *Mi14-3-3* genes may have evolved from the copying of these genes. In addition, 11, 7 and 14 orthologous duplicated gene pairs were found between mango and Arabidopsis, Citrus sinensis and apple (Figure 7), respectively. In Figure 7, two genes connected by a line are defined as homologues; single chromosomes from the two genomes are mostly joined by lines of the same colour, indicating evolutionary similarities between those genomes. Most of the *14-3-3* genes were conserved during polyploidy, indicating their evolutionary importance. Therefore, a homology analysis provided a new way to study the evolutionary characteristics of *Mi14-3-3* genes.

### 2.8. Expression Profiles of Mi14-3-3 Genes in Different Tissues

**Figure 8:**
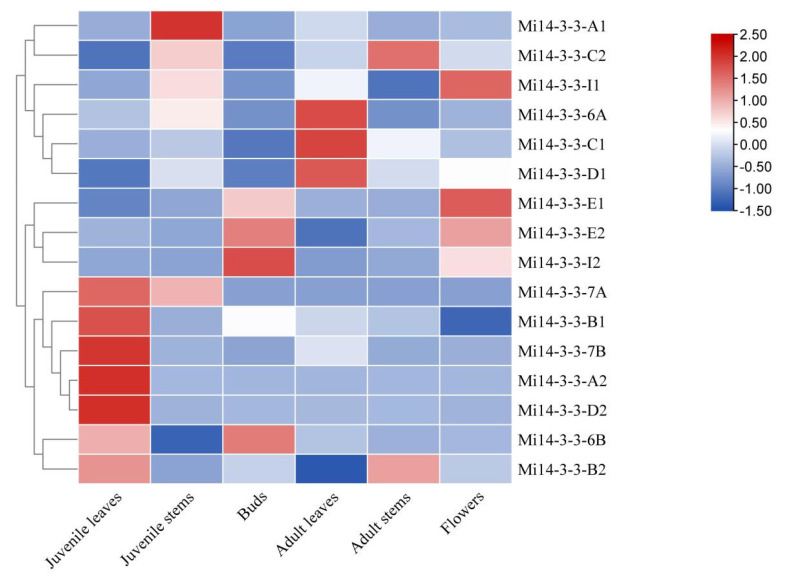
Expression analysis of *Mi14-3-3s* in different tissues and organs of mango plants. Shown is a heatmap generated by TBtools showing a cluster map of the *Mi14-3-3* genes in different tissues. The colour gradient (red/white/blue) indicates the gene expression level (from high to low).

The expression patterns of the 16 *14-3-3* genes in different tissues and organs of mango were analysed. The results showed that the expression patterns of the *14-3-3* gene family members were different in different tissues (Figure 8). *Mi14-3-3-E1* was expressed in most mango tissues, and *Mi14-3-3-E1* was expressed in growing tissue. *Mi14-3-3-I1* was highly expressed in the buds and flowers, which suggests that *Mi14-3-3-I1* might play an important role in the flowering and development of mango. *Mi14-3-3-A2* and *Mi14-3-3-D2* were expressed mostly in young leaves. *Mi14-3-3-6B* and *Mi14-3-3-I2* were expressed mostly in buds, and their expression was very low in the other tissues, suggesting that these two genes might play specific roles in flower bud development.

### 2.9. Expression Patterns of Mi14-3-3s under Abiotic Stress

**Figure 9:**
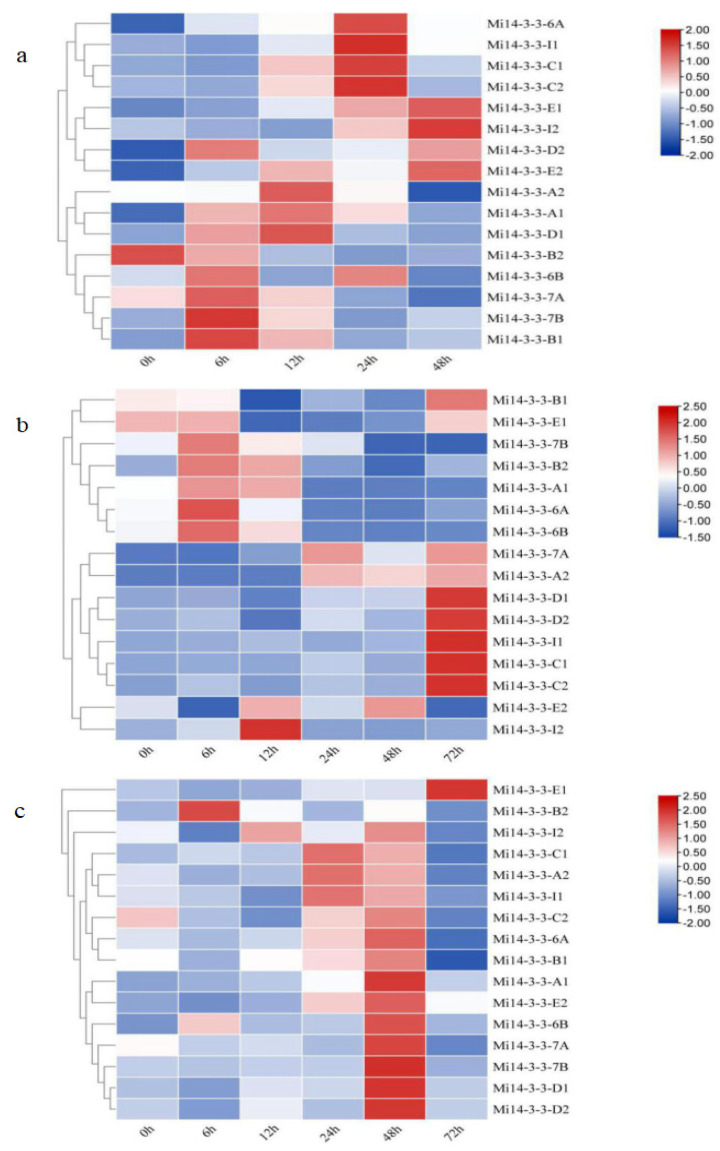
Expression profiles of *Mi14-3-3s* in response to different stresses. (**A**) Cold stress (2 °C). (**B**) Treatment with 30% PEG6000, simulating drought stress. (**C**). NaCl (300 mM) treatment. The relative expression was normalized to that of the *ACTIN* gene, used as a reference, via the 2^−∆∆Ct^ method. The heatmaps were generated based on the log2 (treatment expression/control expression) values with HemI. The colour gradient (red/white/blue) indicates the gene expression level (from high to low). The values indicate the means of three biological replications.

Drought, soil salinization and low temperature are the main natural factors affecting mango yield. Therefore, we used low temperature (2 °C) stress, 30% polyethylene glycol 6000 (PEG6000)-simulated drought stress and 300 mM salt stress to further explore the expression patterns of *Mi14-3-3s* under abiotic stress via quantitative real-time PCR (qRT–PCR). We found that different *Mi14-3-3* gene pairs had different expression patterns under abiotic stress. The expression patterns of the *Mi14-3-3-E1/E2*, *Mi14-3-3-7B/B1*, and *Mi14-3-3-6A* genes were upregulated at all time points during cold treatment (Figure 9A). The expression levels of four genes, *Mi14-3-3-A1*, *Mi14-3-3-6B*, *Mi14-3-3-I1*, and *Mi14-3-3-I2*, showed dynamic changes during cold treatment, while the expression levels of *Mi14-3-3-D1/D2* and *Mi14-3-3-C1/C2* did not change significantly after cold treatment. In contrast, *Mi14-3-3-B2* and *Mi14-3-3-7A/A2* were significantly downregulated under cold treatment. Under drought stress, the expression of *Mi14-3-3-6B/B2*, *Mi14-3-3-6A/A1*, and *Mi14-3-3-I2* increased significantly at 12 h and then decreased (Figure 9B). The expression levels of seven genes (*Mi14-3-3-A2*, *Mi14-3-3-7A*, *Mi14-3-3-C1*, *Mi14-3-3-C2*, *Mi14-3-3-D1*, *Mi14-3-3-D2*, and *Mi14-3-3-I1*) were generally downregulated at one time point. Five of the sixteen Mi14-3-3 genes (*Mi14-3-3-7A*, *Mi14-3-3-A2*, *Mi14-3-3-D1*, *Mi14-3-3-E1*, and *Mi14-3-3-I1*) were expressed in the same way; their expression increased first and then decreased under salt stress (Figure 9B). The expression of 11 genes (*Mi14-3-3-6A*, *Mi14-3-3-A1*, *Mi14-3-3-6B*, *Mi14-3-3-7B*, *Mi14-3-3-B1*, *Mi14-3-3-B2*, *Mi14-3-3-C1*, *Mi14-3-3-C2*, *Mi14-3-3-D2*, *Mi14-3-3-E2*, and *Mi14-3-3-I2*) did not change significantly under salt stress, but the expression of *Mi14-3-3-6A/6B/7B/B1/B2*, *Mi14-3-3-A1*, *Mi14-3-3-D2*, *Mi14-3-3-E2*, *Mi14-3-3-C1/C2*, and *Mi14-3-3-I2* decreased with time.

## 3. Discussion

As regulatory factors involved in plant hormone signal transduction and metabolism, 14-3-3 proteins play important roles in plant growth, development and stress responses. Using a bioinformatic method, we identified 16 members of the *14-3-3* gene family in mango. In marked contrast to the findings of previous studies on *Arabidopsis thaliana* [19], rice [20] and apple [24], the Mi*14-3-3* family genes were not evenly distributed across chromosomes. The analysis of the physicochemical properties of the Mi14-3-3 proteins showed that the proteins were acidic and thus stable, similar to apple 14-3-3 proteins. The secondary structures of Mi14-3-3 proteins were similar to those of Arabidopsis and apple 14-3-3 proteins. In addition, the three-dimensional structures of Mi14-3-3 family proteins were very similar to those of Arabidopsis 14-3-3 family proteins, and the Mi*14-3-3* gene family members could be classified into ε and non-ε classes according to their phylogenetic relationships. The Mi14-3-3 proteins were most closely related to apple 14-3-3 proteins, which is consistent with the fact that mango and apple are woody plant species. A genetic structure analysis showed that ε class *Mi14-3-3* genes contained more exons and introns than the non-ε class *Mi14-3-3* genes did, suggesting that evolution drove this diversity. In other species, the non-ε class 14-3-3 proteins are more abundant than the ε class 14-3-3 proteins, while in mango, the numbers of 14-3-3 proteins were the same. Conserved motif analysis showed that there were seven conserved motifs in the ε class and non-ε class *Mi14-3-3s*. The C-terminal motifs were highly variable; these motifs directly affect the interactions between 14-3-3 proteins and other proteins. The diverse motifs in the *Mi14-3-3* gene family are the core 14-3-3 protein structures that bind to many ligands [28]. Gene families evolve from tandem and fragmented duplication of genes. Through a homology analysis, tandem and fragment repeats were found in *Mi14-3-3* genes, indicating the possibility of horizontal duplication within the *Mi14-3-3* gene family [29].

The promoters of the *Mi14-3-3* genes were found to contain several light-responsive cis-regulatory elements, which implies that the *Mi14-3-3* genes are induced by light signals and participate in a complex light signal response. In Arabidopsis thaliana, 14-3-3 protein 1 (Phot1) is specifically bound under blue light [30], and under red light, *At14-3-3μ* and *At14-3-3ν* interact with photoperiod regulatory proteins [31]. The *Mi14-3-3* gene promoters contained several cis-regulatory elements that respond to abscisic acid (ABA), GA, auxin and other signals. Studies on barley *Hv14-3-3* have shown that 14-3-3 proteins are induced by ABA signalling and participate in ABA signalling [32]. By regulating the subcellular location of *REPRESSION OF SHOOT GROWTH* (*RSG*) transcription factors, tobacco 14-3-3 proteins negatively regulate GA expression [33]. In Arabidopsis, 14-3-3 proteins can also maintain ethylene levels by increasing the stability of 1-aminocyclopropane-1-carboxylate synthase (ACS) proteins and reducing E3 ubiquitin ligase binding [34]. In addition, Arabidopsis ε class 14-3-3 proteins affect the polarity-regulated indole-acetic acid (IAA) concentration gradient of the PIN-FORMED auxin efflux protein on the plasma membrane, thereby participating in the plant development process regulated by IAA [35].

The expression pattern of *Mi14-3-3-E1* was different in different tissues. *Mi14-3-3-7A* was expressed mostly in the stems and leaves, *Mi14-3-3-I1* was expressed mostly in the flower buds and flowers, and *Mi14-3-3-6B* and *Mi14-3-3-I2* were expressed only in the flower buds. The homologous gene of *Mi14-3-3-6B* in Arabidopsis is *14-3-3*Ω. With *14-3-3*Ω acting as a universal regulator, *SUPPRESSOR OF OVEREXPRESSION OF CONSTANS1* (*SOC1*) regulates the flower-forming factors *FT* and *FD*, which induce the accumulation of the oxidative stress factor *Oxs2* and the expression of the flower-forming factor *FT*, both of which are involved in early flowering in Arabidopsis. The accumulation of *Oxs2* in the cytoplasm inhibits *FT* entry into the nucleus and inhibits early flowering in Arabidopsis [36]. In the GA pathway, the *bZIP* transcription factor *RSG*, which is a key enzyme involved in GA synthesis in plants, has been shown to interact with *14-3-3s* via yeast two-hybrid screening. In tobacco and Arabidopsis, GA promotes plant flowering. 14-3-3 proteins have been shown to bind to *RSGs* phosphorylated by *NTCDPK1*, preventing those *RSGs* from entering the nucleus and thus negatively regulating endogenous GA synthesis-related signalling [33,37,38].

Cold stress decreased the transcript levels of *Mi14-3-3s* in the leaves, and two of the promoters contained *LTRs*. *Arabidopsis RCI1A* (*AtGRF3*) upregulates ethylene synthesis and inhibits ethylene-dependent cold tolerance-related gene expression under cold stress [9]. The *AtGRF3* homologous gene *Mi14-3-3-A2* in mango was downregulated under cold stress, which suggests that *Mi14-3-3-A2* might be a positive regulator of cold stress. Under normal growth conditions or drought stress conditions, *OsGF14B* can alter the levels of stress-related parameters and the expression of stress-related genes. Through ABA signalling, *OsGF14B* participates in and negatively regulates both the drought and the osmotic resistance of rice plants [39]. The expression of *Mi14-3-3-A1*, a homologous gene of *AtGRF7* in Arabidopsis thaliana, was initially upregulated during drought treatment, but it was downregulated after 12 h of drought treatment. The expression of the *Mi14-3-3-A1* homolog *AtGRF7* in *Arabidopsis thaliana* has been found to be increased in a mutant in which *AtGRF7* was silenced [10]; however, the expression of *Mi14-3-3-A1* in the current study decreased after 12 h of drought treatment. Under salt stress, the *GhGRF3*, *GhGRF4*, *GhGRF5*, *GhGRF7* and *GhGRF16* genes have been found to be downregulated in the leaves of cotton [18], and *GhN/AINV13* has been shown to interact with 14-3-3 proteins to improve resistance to stresses, such as salt stress in upland cotton [40]. In the present study, five *14-3-3* genes, *Mi14-3-3-7A*, *Mi14-3-3-A2*, *Mi14-3-3-D1*, *Mi14-3-3-E1*, and *Mi14-3-3-I1*, were upregulated or downregulated after salt treatment, which indicates that *Mi14-3-3* genes are controlled by different regulatory mechanisms under stress. Under drought stress, overexpression of *14-3GF* (which encodes a 14-3-3 protein) in maize promotes maize symbiosis and resistance to stress from arbuscular mycorrhizae [41]. Gene expression analysis has shown that *ZmGF14-6* of maize (which encodes a 14-3-3 protein) is upregulated in response to fungal infection and salt treatment, but it is downregulated in response to drought stress [42]. Moreover, the drought tolerance of *Arabidopsis thaliana* mutant progeny is improved by silencing the *14-3-3* protein-encoding gene *AtGF14m* [10]. In transgenic *SiGRF1*-overexpressing plants under high-salinity conditions, the gene expression levels of the flowering genes *FT* and *LEAFY* (*LFY*) increase, resulting in early flowering [43]. In addition, the expression levels of the *14-3-3* protein-encoding gene *MdGRF11* in apple increase significantly under salt stress and low temperature stress [44].

## 4. Materials and Methods

### 4.1. Materials and Treatments

The information on the *Mi14-3-3* gene family in this study was based on the sequenced variety ‘Sijimi’, which flowers more than once a year. This variety was developed by researchers at Guangxi University and has high breeding and scientific research value. In this experiment, mango plants with mature and stable characteristics were cultivated in the experimental garden of Guangxi University; the flowering habits of these plants contrast with the flowering habits of annuals and other woody plant species. Tissue and organ samples of mango were collected from mature leaves, mature stems, and flowers of mature trees, while leaves, stems, and buds were collected from young trees. For the abiotic stress experiments, 10-year-old Mangifera indica plants were used as samples. These trees were subjected to low temperature (2 °C) stress, 30% PEG6000-simulated drought stress and 300 mM salt stress. Samples were collected before and after 6 h, 12 h, 24 h, 48 h and 72 h of treatment. The samples were immediately frozen and stored at -80 °C until use.

### 4.2. Identification and Sequence Analysis of 14-3-3 Gene Family Members in Mango

To identify potential *14-3-3* gene family members in the mango genome, the ‘SiJiMi’ mango genome (unpublished) was used. The sequences of 15 members of the *14-3-3* family were obtained from the *Arabidopsis thaliana* genome database [45] and were used as query sequences to search for sequences of *Mi14-3-3* homologous genes in the mango genome database via local BLAST searches. After screening according to an e value < 1 × 10^−10^ and an identity > 40%, we used the Pfam [46] (https://pfam.janelia.org, accessed on 6 January 2021) and SMART [47] (http://SMART.emblheidelberg.de, accessed on 2 March 2021) online tools to identify the domains of the retrieved Mi14-3-3 protein sequences and eliminate the proteins with missing domains. Finally, the candidate genes of the *Mi14-3-3* family were obtained. In addition, the online ExPASy program (http://www.ExPASy.org/tools, accessed on 12 March 2021) was used to predict the three parameters (length, MW and pI) of each 14-3-3 protein [48]. The amino acid number, MW and theoretical pI of each Mi14-3-3 protein were predicted by ProtParam [49]. The subcellular localization of each member was predicted using the subcellular localization prediction tool WoLF PSORT (https://wolfpsort.hgc.jp, accessed on 20 March 2021), and SWISS-MODEL (https://swissmodel.expasy.org, accessed on 14 March 2021) was used to construct homologous models of the proteins and obtain models of their three-dimensional structures.

### 4.3. Chromosomal Location and Gene Structure Analyses

The chromosomal locations of the *Mi14-3-3* gene family members were extracted from the gff3 file of the mango genome annotation, and a map of the chromosomal gene distribution was constructed with MapChart software [50]. Using the Gene Structure Display Server (GSDS) [51] online tool (http://GSDS.cbi.pku.edu.cn, accessed on 3 April 2021), we predicted the numbers of exons and introns, and the *14-3-3* gene structures were determined. The conserved amino acid sequences of the proteins were analysed online via MEME software (http://MEME-suite.org, accessed on 5 April 2021); the number of motifs was set to 10, and the other parameters were set to their default values [52]. The motif structures were drawn with TBtools [53].

### 4.4. Sequence Alignment and Phylogenetic Analyses

The amino acid conservation of the protein sequences was analysed with DNAMANx software. The amino acid sequences of Arabidopsis, rice and apple 14-3-3 proteins were downloaded from the Arabidopsis, rice and apple genome databases, respectively, and the online tool CLUSTAL OMEGA (https://www.ebi.ac.uk/Tools/msa/clustalo, accessed on 9 April 2021) was used for multiple sequence alignment of the 14-3-3 proteins of the three plant species [54]. A phylogenetic tree was constructed by the adjacency method in MEGA 11.0 (bootstrap number set to 1000) [55].

### 4.5. Prediction of Cis-Regulatory Elements

The sequence of the 2 kb region upstream of the start codon of each *Mi14-3-3* gene was extracted from the gff3 file of the mango genome annotation and considered the promoter sequence [56]. Predictions of cis-regulatory elements within the promoter were then made via the PlantCARE database (http://bioinformatics.psb.ugent.be/webtools/plantcare/html, accessed on 5 March 2021).

### 4.6. Tandem Duplication and Synteny Analysis

Tandem duplication and synteny relationships were analysed using Circos 0.63 (http://circos.ca, accessed on 18 April 2021) [57]. According to previously published papers, gene duplication events were defined by their chromosomal locations: genes on the same chromosome were considered tandem repeat genes, and genes located on different chromosomes were considered segmental repeat genes [58,59]. A homology analysis of apple, *Arabidopsis thaliana* and citrus was carried out using a plant genome replication database (http://chibba.agtec.uga.edu/duplication, accessed on 11 May 2021), and TBtools was used to display linked pairs [53].

### 4.7. RNA Extraction and qRT–PCR

An improved nucleic acid extraction method was used to extract RNA from mango. The mango RNA was extracted with an Easy Pure^®^ Plant RNA Kit (TransGen Biotech, Beijing, China). Reverse transcription was performed using the reverse transcription enzyme PrimeScript^TM^ Reverse Transcriptase II (Takara) according to the manufacturer’s instructions. The cDNA was then concentrated spectrophotometrically to 50 ng/L. Based on the sequence information of the *14-3-3* genes and the mango internal reference gene *MiACT1*, primers for each gene were designed with Primer 3 Plus online software. qRT–PCR was performed with an ABI7500 instrument (Applied Biosystems, Foster, USA) according to the manufacturer’s instructions. Each sample was divided into 3 replicates. The data were processed by the 2^−ΔΔCT^ method [60], and a heatmap of the gene expression profile was constructed with TBtools software [53].

## 5. Conclusions

In summary, mango *14-3-3* genes are largely involved in plant development and stress responses, but the roles of the different members in stress responses and in signal transduction need to be further studied. In this study, 16 members of the *Mi14-3-3* gene family were identified by bioinformatic analysis, and the results of evolutionary analysis revealed that the mango *14-3-3* genes have undergone duplication and loss throughout evolution. Taken together, the results show that the members of the *Mi14-3-3* gene family might be involved in plant growth, development, stress responses and other physiological and biochemical processes; therefore, further study of the function and regulation of mango *14-3-3* family members is very important.

## Figures and Tables

**Table 1:** Information concerning *14-3-3* genes in mango.

Gene Name	Gene Locus	Chr	aa	pI	II	MW (Da)	Gene Entry Number
*Mi14-3-3-A1*	15934509- 15936351	Chr11	257	4.78	47.14	29,051.64	OK491862
*Mi14-3-3-A2*	8935256- 8937263	Chr12	262	4.74	48.11	29,631.29	OK491863
*Mi14-3-3-B1*	4489787- 4491126	Chr10	252	4.83	37.22	28,431.38	OK491864
*Mi14-3-3-B2*	3572447- 3574125	Chr19	252	4.79	39.12	28,503.45	OK491865
*Mi14-3-3-C1*	4366341- 4369386	Chr16	265	4.80	35.13	29,899.64	OK491866
*Mi14-3-3-C2*	9746038- 9747732	Chr9	247	4.85	41.86	28,078.73	OK491867
*Mi14-3-3-D1*	18226835- 18229518	Chr6	257	4.77	46.99	29,284.95	OK491868
*Mi14-3-3-D2*	1716202- 1718844	Chr13	257	4.68	45.77	29,422.24	OK491869
*Mi14-3-3-E1*	3882198- 3883306	Chr19	261	4.68	44.49	29,583.16	OK491870
*Mi14-3-3-E2*	4744386- 4745564	Chr10	261	4.69	51.96	29,607.16	OK491871
*Mi14-3-3-I1*	2799060- 2800971	Chr4	278	4.83	49.60	30,968.14	OK491872
*Mi14-3-3-I2*	8264404- 8266523	Chr8	267	4.93	51.64	30,230.90	OK491873
*Mi14-3-3-6A*	364937- 367380	Chr7	261	4.72	48.41	29,541.14	OK203791
*Mi14-3-3-6B*	12389595- 12391788	Chr4	261	4.76	50.54	29,376.81	OK203792
*Mi14-3-3-7A*	4859531- 4862214	Chr10	266	5.01	42.85	30,506.27	OK491860
*Mi14-3-3-7B*	4027530- 4010130	Chr19	254	4.85	45.50	28,807.40	OK491861

## Data Availability

Not applicable.

## Supplementary Materials

The following are available online at https://www.mdpi.com/article/10.3390/ijms23031593/s1.
