# Supplementary material for: Genome-Wide Identification and Expression Analysis of the 14-3-3 Gene Family in Mango (Mangifera indica L.)
Source: Int J Mol Sci. 2022 Jan 29;23(3):1593. doi: 10.3390/ijms23031593 (PMC8835932; doi:10.3390/ijms23031593)
Supplement: Supplementary file 1 [file ijms-23-01593-s001.zip › ijms-1566018-supplementary/Table S1.pdf]

Table S1 Mango 14-3-3 protein secondary structure main component ratio

Protein	α-helix	β-turn	Random coil	Extended strand
Mi14-3-3-A1	68.09%	1.95%	22.18%	7.78%
Mi14-3-3-A2	69.47%	1.91%	21.76%	6.87%
Mi14-3-3-B1	68.65%	1.59%	21.83%	7.94%
Mi14-3-3-B2	73.81%	1.19%	19.05%	5.95%
Mi14-3-3-C1	65.28%	1.51%	25.28%	7.92%
Mi14-3-3-C2	70.04%	2.02%	20.24%	7.69%
Mi14-3-3-D1	69.26%	0.78%	22.57%	7.39%
Mi14-3-3-D2	68.48%	0.78%	22.96%	7.78%
Mi14-3-3-E1	69.73%	1.15%	22.22%	6.90%
Mi14-3-3-E2	67.82%	1.53%	23.37%	7.28%
Mi14-3-3-I1	66.19%	1.44%	23.38%	8.99%
Mi14-3-3-I2	67.04%	0.75%	25.09%	7.12%
Mi14-3-3-6A	70.11%	3.07%	19.92%	6.90%
Mi14-3-3-6B	70.50%	2.30%	20.31%	6.90%
Mi14-3-3-7A	66.54%	1.13%	23.31%	9.02%
Mi14-3-3-7B	68.11%	0.79%	23.23%	7.87%
