# Supplementary material for: Genome-Wide Identification and Expression Analysis of the 14-3-3 Gene Family in Mango (Mangifera indica L.)
Source: Int J Mol Sci. 2022 Jan 29;23(3):1593. doi: 10.3390/ijms23031593 (PMC8835932; doi:10.3390/ijms23031593)
Supplement: Supplementary file 1 [file ijms-23-01593-s001.zip › ijms-1566018-supplementary/The conserved motifs among Mi14-3-3 proteins.pdf]

The conserved motifs among Mi14-3-3 proteins.

	Logo	E-value	Sites	Width
1.	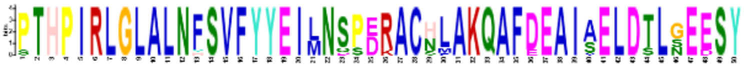	1.8e-629	16	50
2.	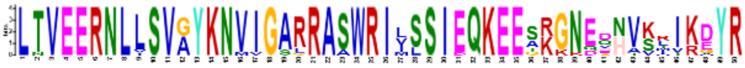	9.1e-536	16	50
3.	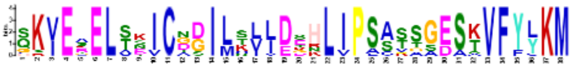	4.8e-310	16	38
4.	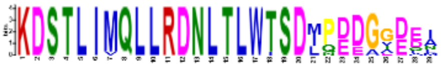	3.7e-264	14	29
5.	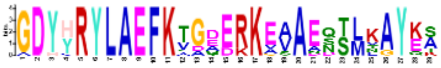	3.1e-241	16	29
6.	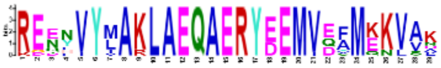	6.9e-234	15	29
7.	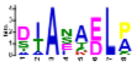	5.7e-013	16	8
8.	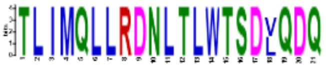	2.9e-004	2	21
9.	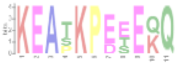	8.0e-001	4	11
10.	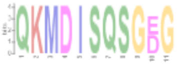	6.8e+000	2	11
